# Dietary fat, cholesterol and colorectal cancer in a prospective study

**DOI:** 10.1054/bjoc.2001.1906

**Published:** 2001-08

**Authors:** R Järvinen, P Knekt, T Hakulinen, H Rissanen, M Heliövaara

**Affiliations:** Department of Clinical Nutrition, University of Kuopio, P.O. Box 1627, Kuopio, FIN-70211, Finland; National Public Health Institute, Mannerheimintie 166, Helsinki, FIN-00300, Finland; The Social Insurance Institution, Helsinki and Turku, Finland; Finnish Cancer Registry, Liisankatu 21 B, Helsinki, FIN-00170, Finland; Unit of Cancer Epidemiology, Karolinska Institute, Radiumhemmet, Stockholm, Sweden

**Keywords:** colorectal neoplasms, dietary cholesterol, eggs, fats, meat, protein

## Abstract

The relationships between consumption of total fat, major dietary fatty acids, cholesterol, consumption of meat and eggs, and the incidence of colorectal cancers were studied in a cohort based on the Finnish Mobile Clinic Health Examination Survey. Baseline (1967–1972) information on habitual food consumption over the preceding year was collected from 9959 men and women free of diagnosed cancer. A total of 109 new colorectal cancer cases were ascertained late 1999. High cholesterol intake was associated with increased risk for colorectal cancers. The relative risk between the highest and lowest quartiles of dietary cholesterol was 3.26 (95% confidence interval 1.54–6.88) after adjusting for age, sex, body mass index, occupation, smoking, geographic region, energy intake and consumption of vegetables, fruits and cereals. Consumption of total fat and intake of saturated, monounsaturated, or polyunsaturated fatty acids were not significantly associated with colorectal cancer risk. Nonsignificant associations were found between consumption of meat and eggs and colorectal cancer risk. The results of the present study indicate that high cholesterol intake may increase colorectal cancer risk, but do not suggest the presence of significant effects of dietary fat intake on colorectal cancer incidence. © 2001 Cancer Research Campaign http://www.bjcancer.com


British Journal of Cancer (2001) 85(3), 357–361
© 2001 Cancer Research Campaign
doi: 10.1054/ bjoc.2001.1906, available online at http://www.idealibrary.com on IDEAL®

http://www.bjcancer.com

### Dietary fat, cholesterol and colorectal cancer in a prospective study

**R Järvinen[1], P Knekt[2,3], T Hakulinen[4,5], H Rissanen[2] and M Heliövaara[2,3]**

[1]Department of Clinical Nutrition, University of Kuopio, P.O. Box 1627, FIN-70211 Kuopio, Finland; [2]National Public Health Institute, Mannerheimintie 166, FIN-00300 Helsinki, Finland; [3]The Social Insurance Institution, Helsinki and Turku, Finland; [4]Finnish Cancer Registry, Liisankatu 21 B, FIN-00170 Helsinki, Finland; [5]Unit of Cancer Epidemiology, Radiumhemmet, Karolinska Institute, Stockholm, Sweden

**Summary** The relationships between consumption of total fat, major dietary fatty acids, cholesterol, consumption of meat and eggs, and the incidence of colorectal cancers were studied in a cohort based on the Finnish Mobile Clinic Health Examination Survey. Baseline (1967–1972) information on habitual food consumption over the preceding year was collected from 9959 men and women free of diagnosed cancer. A total of 109 new colorectal cancer cases were ascertained late 1999. High cholesterol intake was associated with increased risk for colorectal cancers. The relative risk between the highest and lowest quartiles of dietary cholesterol was 3.26 (95% confidence interval 1.54–6.88) after adjusting for age, sex, body mass index, occupation, smoking, geographic region, energy intake and consumption of vegetables, fruits and cereals. Consumption of total fat and intake of saturated, monounsaturated, or polyunsaturated fatty acids were not significantly associated with colorectal cancer risk. Nonsignificant associations were found between consumption of meat and eggs and colorectal cancer risk. The results of the present study indicate that high cholesterol intake may increase colorectal cancer risk, but do not suggest the presence of significant effects of dietary fat intake on colorectal cancer incidence. © 2001 Cancer Research Campaign http://www.bjcancer.com

**Keywords**: colorectal neoplasms; dietary cholesterol; eggs; fats; meat; protein

Development of colorectal cancer has been suggested to be closely related to environmental exposures, especially those arising from diet. High dietary fat, protein (especially animal fat and protein), and consumption of meat have been frequently associated with increased incidence of large bowel cancers in ecological comparisons between and within countries (Armstrong and Doll, 1975; Rose et al, 1986). Results from animal studies also support the significance of fat intake in colon cancer development (Lipkin et al, 1999). Nevertheless, findings of epidemiologic studies on the relationship between fat consumption and colorectal cancer risk have been variable (Potter, 1996; Giovannucci and Goldin, 1997; Word Cancer Research Fund and American Institute for Cancer Research, 1997). High consumption of meat, especially red meat, was associated with elevated colorectal cancer risk in several case–control studies (Word Cancer Research Fund and American Institute for Cancer Research, 1997) and in some (Willett et al, 1990; Giovannucci et al, 1994; Singh and Fraser, 1998) but not in other prospective studies (Bostick et al, 1994; Goldbohm et al, 1994; Gaard et al, 1996; Kato et al, 1997; Pietinen et al, 1999).

Diets containing high amounts of animal fat also provide high amounts of cholesterol. It has been suggested that high cholesterol intake may be a risk factor for colorectal cancers (Cruse et al, 1979; Steinmetz and Potter, 1994). A recent combined analysis of case–control studies found significantly elevated risk for colorectal cancers associated with high cholesterol intake (Howe et al, 1997). Previous prospective studies have reported nonsignificant associations between dietary cholesterol and colorectal cancer occurrence (Willett et al, 1990; Bostick et al, 1994; Giovannucci et al, 1994; Kato et al, 1997; Pietinen et al, 1999). In the present study we investigated the relationships between consumption of dietary fat, cholesterol, protein, meat and eggs and the incidence of colorectal cancer in a cohort based on the Finnish Mobile Clinic Health Examination Survey.

*Received 15 November 2000*
*Revised 2 April 2001*
*Accepted 20 April 2001*

*Correspondence to:* R Järvinen

### POPULATION AND METHODS

The subjects of this study were men and women who participated in a population-based health examination survey carried out in Finland during 1966–1972. A mailed invitation was sent to 33 382 men and 29 058 women who were selected to represent 30 populations from 6 regions of the country, and 81.9% of men and 83.2% of women participated in the examination. Approximately 1 in 5 of the participants were also interviewed for their total habitual diet at baseline started in 1967. Dietary data were collected for 9959 participants who were without diagnosed cancer at baseline. Identification of incident cancer cases during the follow-up until late 1999 was ascertained through the national Finnish Cancer Registry (Teppo et al, 1994). A total of 109 new colorectal cancer cases (63 in colon and 46 in rectum) were recognized as far as late 1999 (International Classification of Diseases, seventh revision, codes 153–154) (World Health Organization, 1955). The total number of men ($n$ = 54) and women ($n$ = 55) who developed colorectal cancer was approximately similar but the number of men with rectal cancer ($n$ = 25) was slightly higher and the number with colon cancer ($n$ = 29) lower than those of women.

Examinees who participated in the diet study were interviewed for their habitual food consumption during the preceding year. A structured questionnaire listing more than 100 different foods and mixed dishes was used to guide the interview carried out by interviewers who were trained home economic teachers and students in nutrition. Several of the questions were open-ended, requiring that they be specified by the respondent during the interview. Consumption of foods could be expressed per day, week, month, or year according to the choice of the respondent. Furthermore, foods whose consumption varied greatly between seasons could be estimated separately for different seasons. Food models made of plastic and rubber as well as samples of real food were available to aid in estimation of the amounts of foods consumed.

The average daily consumption of different food items and nutrients over all food items was computed utilizing a software package specifically developed for this study. The program utilized a recipe file that was compiled from recipes of mixed dishes available at the time of the baseline study. A food composition database to estimate the intake of nutrients over all food items was constructed based on Finnish food composition tables (Rastas et al, 1989). The fatty acid composition data were completed using analysed values in Finnish foods. More specific description of the dietary survey method and reproducibility of food and nutrient consumption at short-term and long-term interval were given in a previous study (Järvinen et al, 1993). Intraclass correlation coefficients for the intake of total protein, total fat, different major dietary fats and cholesterol varied from 0.65 to 0.80 over an interval of 4–8 months, and from 0.46 to 0.58 over an interval of 4–7 years. Long-term agreement for polyunsaturated fat intake was 0.26.

A self-administered questionnaire mailed with the invitation and checked during the examination provided information on sociodemographic factors, smoking, medical history, and use of drugs and food supplements. Weight and height in light indoor clothing without shoes were measured during the examination, and body mass index (kg $m^{-2}$) was calculated.

The associations between dietary variables and the incidence of colon and rectal cancers were investigated by utilizing the Cox proportional hazards model (Cox, 1972). The relative risks (RR) and 95% confidence intervals (CI) were calculated for different quartiles of food and nutrient intakes, using the lowest quartile as the reference category. Quartile ranges were determined separately for men and women. Age, sex, body mass index, occupation, smoking and geographic region were the nondietary covariates included in the model. Dietary energy intake and consumption of food groups of vegetables (without potatoes), fruits and cereals were added to the model as potential dietary confounders. Age, body mass index, energy intake and consumption of vegetables, fruits and cereals were added as continuous variables in the multivariate model whereas occupation (agricultural, industrial, services, white collar, housewives), smoking (never smoker, ex-smoker, current smoker of pipe or cigar, current smoker of fewer than 15 cigarettes per day, current smoker of 15 or more cigarettes per day) and geographical area (6 regions) were added as categorical variables. Age- and sex-adjusted mean intakes of foods and nutrients were estimated based on the general linear model.

### RESULTS

At baseline colorectal cancer cases were older and their body mass index was significantly higher than that of the noncases (Table 1). The mean intake of dietary energy, protein and different fatty acids was approximately similar among colorectal cancer cases and noncases, but the cases consumed more eggs and cholesterol than the noncases, however, the differences were not statistically significant.

A slight nonsignificant decreased risk for colorectal cancers was suggested at the highest energy intake level (not shown in table).

**Table 1** Selected baseline characteristics and mean daily intake of energy, nutrients and foods among the colorectal cancer cases and noncases

| Variable[a] | Colorectal cancer cases (*n* = 109) | Noncases (*n* = 9850) | *P* value |
|---|---|---|---|
| Age, years[b] | 49.5 | 39.0 | <0.001 |
| Body mass index, kg/m$^2$ | 25.6 | 24.8 | 0.02 |
| Current smoking, % | 31.8 | 35.3 | 0.42 |
| Dietary energy, kcal | 2516 | 2595 | 0.32 |
| Protein, g | 93.9 | 93.8 | 0.97 |
| Total fat, g | 109.1 | 109.1 | 0.99 |
| Saturated fatty acids, g | 61.6 | 61.6 | 0.99 |
| Monounsaturated fatty acids, g | 35.3 | 35.2 | 0.94 |
| Polyunsaturated fatty acids, g | 7.6 | 7.7 | 0.79 |
| Cholesterol, mg | 506 | 488 | 0.34 |
| Meat and meat products, g | 153.9 | 145.3 | 0.34 |
| Eggs, g | 36.2 | 34.1 | 0.47 |

[a]Adjusted for age and sex. [b]Adjusted for sex.

The relative risk adjusted for age, sex, body mass index, occupation, smoking and geographical area was 0.78 (95% confidence interval (CI) 0.42–1.44) for colorectal cancers, 0.74 (95% CI = 0.32–1.71) for colon cancer, and 0.82 (95% CI = 0.33–2.04) for rectal cancer. In multivariate analysis including nondietary factors and potential dietary confounders the intakes of protein, total fat, and different major fatty acids were not significantly associated with colorectal cancer risk (Table 2), nevertheless, higher intake levels of total protein and monounsaturated fat were associated with a suggested increased risk of colorectal cancers.

High cholesterol intake predicted an elevated risk for colorectal cancers (Table 2). In multivariate analysis the relative risk for colorectal cancers was 3.26 (95% CI = 1.54–6.88) between the highest and lowest quartiles of dietary cholesterol. Significantly elevated risks were found for both colon (RR = 3.20, 95% CI = 1.21–8.48) and rectal (RR = 3.36, 95% CI = 1.05–10.80) cancers. The relative risk for colorectal cancers adjusted for non-dietary factors was 1.60 (95% CI = 0.92–2.79) and accordingly the higher relative risk in the final model was due to adjustment for energy intake. The relationship between dietary cholesterol intake and colorectal cancer risk persisted after further adjustment for total fat intake. The relative risk between the highest and lowest quartiles of cholesterol intake was 2.98 (95% CI = 1.39–6.39) in multivariate analysis including total fat intake (not shown in table).

Total consumption of meat and meat products, red meat, or liver was not significantly associated with the incidence of colorectal cancers (Table 3). Those who consumed poultry meat had an increased risk for colorectal cancers mainly due to an increased risk for colon cancer. Egg consumption was not significantly related to colorectal cancer risk. Nonsignificant associations were similarly found when we separately investigated eggs prepared by different cooking methods (not shown in table). The relative risk for colorectal cancers in the highest vs. lowest quartiles of fried egg consumption was 1.45 (95% CI = 0.82–2.55); the respective relative risk for consumption of boiled eggs was 1.01 (0.60–1.70).

### DISCUSSION

In this prospective study, high cholesterol intake was found to be associated with increased risk of colorectal cancer. The demonstrated increased risk of colorectal cancer was likely to be specific for

© 2001 Cancer Research Campaign

**Table 2** Relative risks (and 95% confidence intervals) for colorectal cancers in quartiles of intakes of dietary energy, protein, total fat, major fatty acids, and cholesterol

| Quartile of daily intake | Quartile ranges | | Colorectal cancer (*n* = 109) RR (95% CI)[a] | Colon cancer (*n* = 63) RR (95% CI)[a] | Rectum cancer (*n* = 46) RR (95% CI)[a] |
|---|---|---|---|---|---|
| | Men | Women | | | |
| Protein (g) | | | | | |
| 1 | <84.7 | <59.2 | 1 | 1 | 1 |
| 2 | 84.7–103.2 | 59.2–72.9 | 2.26 (1.25–4.09) | 1.80 (0.82–3.92) | 3.13 (1.25–7.82) |
| 3 | 103.3–126.5 | 73.0–89.6 | 2.19 (1.08–4.45) | 2.06 (0.83–5.09) | 2.37 (0.75–7.43) |
| 4 | >126.5 | >89.6 | 2.28 (0.86–6.02) | 1.59 (0.44–5.72) | 3.69 (0.82–16.51) |
| Total fat (g) | | | | | |
| 1 | <95.7 | <64.7 | 1 | 1 | 1 |
| 2 | 95.7–121.0 | 64.7–83.0 | 1.47 (0.82–2.66) | 1.51 (0.68–3.33) | 1.44 (0.60–3.49) |
| 3 | 121.1–151.5 | 83.1–105.5 | 1.63 (0.80–3.33) | 1.88 (0.73–4.85) | 1.38 (0.47–4.08) |
| 4 | >151.5 | >105.5 | 1.47 (0.52–4.20) | 1.86 (0.46–7.43) | 1.09 (0.22–5.41) |
| Saturated fatty acids (g) | | | | | |
| 1 | <53.5 | <35.6 | 1 | 1 | 1 |
| 2 | 53.5–68.7 | 35.6–46.6 | 1.09 (0.60–1.99) | 0.92 (0.41–2.07) | 1.37 (0.56–3.36) |
| 3 | 68.8–86.6 | 46.7–60.1 | 1.72 (0.88–3.36) | 1.77 (0.74–4.25) | 1.68 (0.59–4.80) |
| 4 | >86.6 | >60.1 | 1.47 (0.56–3.83) | 1.56 (0.44–5.48) | 1.39 (0.31–6.14) |
| Monounsaturated fatty acids (g) | | | | | |
| 1 | <30.5 | <20.8 | 1 | 1 | 1 |
| 2 | 30.5–38.8 | 20.8–26.7 | 1.52 (0.83–2.77) | 1.44 (0.64–3.25) | 1.63 (0.66–4.02) |
| 3 | 38.9–49.2 | 26.8–34.0 | 2.10 (1.04–4.26) | 2.40 (0.95–6.08) | 1.74 (0.58–5.21) |
| 4 | >49.2 | >34.0 | 2.37 (0.86–6.51) | 2.37 (0.61–9.19) | 2.38 (0.52–10.85) |
| Polyunsaturated fatty acids (g) | | | | | |
| 1 | <5.9 | <4.1 | 1 | 1 | 1 |
| 2 | 5.9–7.7 | 4.1–5.5 | 0.98 (0.57–1.70) | 0.84 (0.40–1.78) | 1.20 (0.54–2.69) |
| 3 | 7.8–10.3 | 5.6–7.5 | 1.12 (0.61–2.04) | 1.31 (0.61–2.82) | 0.84 (0.31–2.26) |
| 4 | >10.3 | >7.5 | 1.13 (0.56–2.26) | 0.97 (0.38–2.46) | 1.35 (0.47–3.89) |
| Cholesterol (mg) | | | | | |
| 1 | <401.6 | <288.3 | 1 | 1 | 1 |
| 2 | 401.6–517.2 | 288.3–380.3 | 1.79 (1.00–3.19) | 1.64 (0.77–3.49) | 2.05 (0.83–5.09) |
| 3 | 517.3–668.0 | 380.4–501.0 | 2.04 (1.07–3.89) | 1.84 (0.79–4.32) | 2.36 (0.87–6.38) |
| 4 | >668.0 | >501.0 | 3.26 (1.54–6.88) | 3.20 (1.21–8.48) | 3.36 (1.05–10.80) |

[a]Relative risk adjusted for age, sex, body mass index, occupation, smoking, geographical area, energy intake and consumption of vegetables, fruits and cereals.

**Table 3** Relative risks (and 95% confidence intervals) for colorectal cancers in quartiles of intakes of meat and eggs

| Quartile of daily intake (g) | Quartile ranges | | Colorectal cancer (*n* = 109) RR (95% CI)[a] | Colon cancer (*n* = 63) RR (95% CI)[a] | Rectum cancer (*n* = 46) RR (95% CI)[a] |
|---|---|---|---|---|---|
| | Men | Women | | | |
| Meat, all | | | | | |
| 1 | <101 | <67 | 1 | 1 | 1 |
| 2 | 101–151 | 67–101 | 1.07 (0.62–1.86) | 0.75 (0.36–1.58) | 1.71 (0.74–3.97) |
| 3 | 152–220 | 102–145 | 1.31 (0.73–2.32) | 1.06 (0.49–2.26) | 1.77 (0.72–4.35) |
| 4 | >220 | >145 | 1.52 (0.78–2.96) | 1.54 (0.66–3.57) | 1.48 (0.49–4.45) |
| Red meat | | | | | |
| 1 | <94 | <61 | 1 | 1 | 1 |
| 2 | 94–141 | 61–92 | 1.06 (0.67–2.01) | 0.71 (0.33–1.51) | 2.18 (0.93–5.10) |
| 3 | 142–206 | 93–134 | 1.55 (0.88–2.73) | 1.29 (0.63–2.66) | 2.11 (0.84–5.28) |
| 4 | >206 | >134 | 1.50 (0.77–2.94) | 1.34 (0.57–3.15) | 1.82 (0.60–5.52) |
| Poultry | | | | | |
| 1 (no) | | | 1 | 1 | 1 |
| 2 (yes) | | | 1.59 (1.04–2.44) | 1.93 (1.12–3.35) | 1.20 (0.60–2.37) |
| Liver | | | | | |
| 1 | <1 | <1 | 1 | 1 | 1 |
| 2 | 1 | 1–2 | 0.84 (0.46–1.51) | 0.79 (0.37–1.69) | 0.92 (0.37–2.30) |
| 3 | 2–5 | 3–5 | 0.94 (0.55–1.58) | 0.96 (0.49–1.88) | 0.90 (0.39–2.08) |
| 4 | >5 | >5 | 1.03 (0.60–1.77) | 0.87 (0.42–1.80) | 1.26 (0.57–2.81) |
| Eggs | | | | | |
| 1 | <17 | <13 | 1 | 1 | 1 |
| 2 | 17–29 | 13–24 | 0.84 (0.49–1.45) | 0.72 (0.35–1.47) | 1.06 (0.45–2.47) |
| 3 | 30–48 | 25–42 | 1.09 (0.65–1.84) | 0.99 (0.50–1.93) | 1.27 (0.56–2.90) |
| 4 | >48 | >42 | 1.22 (0.70–2.13) | 1.02 (0.48–2.14) | 1.54 (0.66–3.64) |

[a]Relative risk adjusted for age, sex, body mass index, occupation, smoking, geographical area, energy intake, and consumption of vegetables, fruits and cereals.

© 2001 Cancer Research Campaign 

dietary cholesterol since the association was independent of dietary fat intake, and important food sources of dietary cholesterol, meat and eggs, showed only nonsignificant relationships with colorectal cancer risk. However, we cannot exclude the possibility that the association suggested for dietary cholesterol was due to other dietary or life-style habits potentially related to cholesterol intake.

Results from animal studies indicate that cholesterol acts as a cocarcinogen in the development of colon cancer. Feeding animals with high-cholesterol diets increased the number of induced colon cancers (Cruse et al, 1978; Hiramatsu et al, 1992) and enhanced the induction and development of colonic preneoplastic lesions in carcinogen-treated animals (Rao et al, 1992). There are several mechanisms by which cholesterol intake could modify the carcinogenic process (Steinmetz and Potter, 1994). However, the effect of cholesterol may be dependent on whether it is given during early or later stages of cancer development. When fed to animals only during the promotion stage, cholesterol may even decrease the appearance of induced colon tumours (Cohen et al, 1982; El-Sohemy et al, 1996).

Findings from previous epidemiological studies on the relationship between dietary cholesterol and colorectal cancer risk have been variable. An ecological study between countries illustrated a close correlation between intake of cholesterol and colon cancer mortality after adjustment for intake of fat and dietary fibre (Liu et al, 1979). A combined analysis of 13 case–control studies from different countries demonstrated a significant direct relationship between dietary cholestrol intake and the occurrence of colorectal cancers (Howe et al, 1997). The findings of previous prospective studies suggested null associations; reported relative risks for colon/colorectal cancers between the highest and lowest cholesterol intake categories varied between 0.91–1.39 (Willett et al, 1990; Bostick et al, 1994; Giovannucci et al, 1994; Kato et al, 1997; Pietinen et al, 1999). The considerably longer follow-up period in the present study (up to 32 years) than in previous studies, in addition to other differences in the study populations, may offer some explanations for the differences in the findings. It may be possible that the period of exposure to dietary cholesterol is important in relation to colorectal cancer development. Due to a long follow-up period we may have been able to detect risk factors that operate at earlier stages of carcinogenesis. It has been suggested that the absolute amounts of cholesterol consumed as a factor in itself might not be as significant as its relationship to the intake of total plant sterols (Nair et al, 1984). Plant sterols may offer protection from cancers by different mechanisms (Awad and Fink, 2000). In the present study population cholesterol intake was relatively high, whereas the intake of phytosterols was apparently low due to especially low consumption of vegetable fats.

Eggs have high contents of cholesterol and can contribute importantly to total cholesterol intake. In epidemiologic studies, findings on the relationship between egg consumption and colorectal cancer have been mixed. Approximately half of the 16 recently reviewed case–control studies reported elevation in the risk of large bowel cancers associated with egg consumption (Word Cancer Research Fund and American Institute for Cancer Research, 1997). Results of a recent large case–control study also addressed the importance of egg consumption in relation to colorectal cancer risk (Le Marchand et al, 1997). One previous prospective study among a low-risk population presented a positive association between egg consumption and the risk of fatal colon cancer during a fairly long follow-up period (Phillips and Snowdon, 1985). In the present study egg consumption was not significantly associated with colorectal cancer risk.

We found no significant associations between the intake of total fat or different major fatty acids and risk of colorectal cancers. These results are in line with the finding from several previous prospective studies (Bostick et al, 1994; Giovannucci et al, 1994; Goldbohm et al, 1994; Gaard et al, 1996; Kato et al, 1997; Pietinen et al, 1999). A combined analysis of 13 case–control studies controlling for total dietary energy intake and dietary cholesterol revealed no significant associations between intake of total fat or any of the major fatty acids and colorectal cancer risk (Howe et al, 1997). However, an elevated colon cancer risk was associated with intake of total fat and animal fat as well as with consumption of saturated and monounsaturated fats in a prospective study of female nurses in the USA (Willett et al, 1990).

Total consumption of protein and meat was not significantly associated with colorectal cancer risk in the present study. Previous prospective studies have also reported nonsignificant associations (Willett et al, 1990; Bostick et al, 1994; Giovannucci et al, 1994; Goldbohm et al, 1994; Pietinen et al, 1999), or decreased risk for colorectal cancers with higher protein intakes (Kato et al, 1997), whereas associations of different protein sources with colon cancer risk have been more variable. Red meat consumption in particular was associated with increased colon cancer risk in large prospective studies from the United States (Willett et al, 1990; Giovannucci et al 1994) but not consistently in other prospective studies (Bostick et al, 1994; Goldbohm et al, 1994; Kato et al, 1997; Pietinen et al, 1999). In addition to fatty acid composition of meat, heterocyclic amines formed during frying and broiling of meat, nitrosamines present in processed meat products or formed in the intestinal contents, and iron provided by meat have been suggested as potential factors which may explain increased colorectal cancer risk with high red meat consumption (Giovannucci and Goldin, 1997). In the present study, the total consumption of meat and red meat showed nonsignificant relationships with colorectal cancer incidence during the follow-up. In our previous studies no significant relationships between consumption of fried meat and colorectal cancer were observed (Knekt et al, 1994), whereas higher consumption of cured meat besides salted and smoked fish, all representing important sources of nitrosamines in the diet, were associated with higher colorectal cancer risk (Knekt et al, 1999).

Prospective study design and comprehensive dietary survey method were obvious advantages in the present study. Due to comprehensive dietary interview method we were able to allow for dietary energy intake which is suggested to be an important confounder in colorectal cancer studies (Giovannucci and Goldin, 1997; Slattery et al, 1997). On the other hand, due to the relatively small number of cancer cases the power of the study to reveal associations could have been diminished. During the long follow-up period dietary habits of participants may have changed. Nevertheless, the results from dietary measurements repeated several years apart suggested moderate agreement on the intake of nutrients under consideration with an exception of polyunsaturated fatty acids (Järvinen et al, 1993).

The findings in this prospective study suggest that high cholesterol intake may be a risk factor for colorectal cancers, whereas no significant relationships were observed between colorectal cancer

*© 2001 Cancer Research Campaign*

incidence and the intake of total fat, various major fatty acids, or total dietary protein.

### ACKNOWLEDGEMENT

This study was supported by a grant from the Swedish Cancer Society.

### REFERENCES

Armstrong B and Doll R (1975) Environmental factors and cancer incidence and mortality in different countries, with special reference to dietary practices. *Int J Cancer* **15**: 617–631

Awad AB and Fink CS (2000) Phytosterols as anticancer dietary components: evidence and mechanism of action. *J Nutr* **130**: 2127–2130

Bostick RM, Potter JD, Kushi LH, Sellers TA, Steinmetz KA, McKenzie DR, Gapstur SM and Folsom AR (1994) Sugar, meat, and fat intake, and nondietary risk factors for colon cancer incidence in Iowa women (United States). *Cancer Causes Control* **5**: 38–52

Cohen BI, Raicht RF and Fazzini E (1982) Reduction of *N*-methyl-*N*-nitrosourea-induced colon tumors in the rat by cholesterol. *Cancer Res* **42**: 5050–5052

Cox DR (1972) Regression models and life-tables (with discussion). *J R Stat Soc B* **34**: 187–220

Cruse JP, Lewin MR, Feruland GP and Clark CG (1978) Co-carcinogenic effects of dietary cholesterol in experimental colon cancer. *Nature* **276**: 822–825

Cruse P, Lewin M and Clark CG (1979) Dietary cholesterol is co-carcinogenic for human colon cancer. *Lancet* **1**: 752–755

El-Sohemy A, Kendall CWC, Rao AV, Archer MC and Bruce WR (1996) Dietary cholesterol inhibits the development of aberrant crypt foci in the colon. *Nutr Cancer* **25**: 111–117

Gaard M, Tretli S and Loken EB (1996) Dietary factors and risk of colon cancer: a prospective study of 50,535 young Norwegian men and women. *Eur J Cancer Prev* **5**: 445–454

Giovannucci E and Goldin B (1997) The role of fat, fatty acids, and total energy intake in the etiology of human colon cancer. *Am J Clin Nutr* **66** (suppl): 1564S–1571S

Giovannucci E, Rimm EB, Stampfer MJ, Colditz GA, Ascherio A and Willett WC (1994) Intake of fat, meat, and fiber in relation to risk of colon cancer in men. *Cancer Res* **54**: 2390–2397

Goldbohm RA, van den Brandt PA, van't Veer P, Brants HAM, Dorant E, Sturmans F and Hermus RJJ (1994) A prospective cohort study on the relation between meat consumption and the risk of colon cancer. *Cancer Res* **54**: 718–723

Hiramatsu Y, Takada H, Yamamura M, Hioki K, Saito K and Yamamoto M (1983) Effect of dietary cholesterol on azoxymethane- induced colon carcinogenesis in rats. *Carcinogenesis* **4**: 553–558

Howe GR, Aronson KJ, Benito E, Castelleto R, Cornee J, Duffy S, Gallagher RP, Iscovich JM, Deng-ao J, Kaaks R, Kune GA, Kune S, Lee HP, Lee M, Miller AB, Peters RK, Potter JD, Riboli E, Slattery ML, Trichopoulos D, Tuyns A, Tzonou A, Watson LF, Whittemore AS, Wu-Williams AH and Shu Z (1997) The relationship between dietary fat intake and risk of colorectal cancer: evidence from the combined analysis of 13 case-control studies. *Cancer Causes Control* **8**: 215–228

Järvinen R, Seppänen R and Knekt P (1993) Short- term and long-term reproducibility of dietary history interview data. *Int J Epidemiol* **22**: 520–527

Kato I, Akhmedkhanov A, Koening K, Toniolo PG, Shore RE and Riboli E (1997) Prospective study of diet and female colorectal cancer: the New York University Women's Health Study. *Nutr Cancer* **28**: 276–281

Knekt P, Steineck G, Järvinen R, Hakulinen T and Aromaa A (1994) Intake of fried meat and risk of cancer: a follow-up study in Finland. *Int J Cancer* **59**: 756–760

Knekt P, Järvinen R, Dich J and Hakulinen T (1999) Risk of colorectal and other gastro-intestinal cancers after exposure to nitrate, nitrite and *N*-nitroso compounds: a follow-up study. *Int J Cancer* **80**: 852–856

Le Marchand L, Wilkens LR, Hankin JH, Kolonel LN and Lyo L-C (1997) A case-control study of diet and colorectal cancer in a multiethnic population in Hawaii (United States): lipids and foods of animal origin. *Cancer Causes Control* **8**: 637–648

Lipkin M, Reddy B, Newmark H and Lamprecht SA (1999) Dietary factors in human colorectal cancer. *Annu Rev Nutr* **19**: 545–586

Liu K, Stamler J, Moss D, Garside D, Persky V and Soltero I (1979) Dietary cholesterol, fat, and fiber, and colon-cancer mortality. An analysis of international data. *Lancet* **2**: 782–785

Nair PP, Turjman N, Kessie G, Calkins B, Goodman GT, Davidovitz H and Nimmagadda G (1984) Diet, nutrition intake, and metabolism in populations at high and low risk for colon cancer. *Am J Clin Nutr* **40**: 927–930

Phillips RL and Snowdon DA (1985) Dietary relationships with fatal colorectal cancer among Seventh-Day Adventists. *J Natl Cancer Inst* **74**: 307–317

Pietinen P, Malila N, Virtanen M, Hartman TJ, Tangrea JA, Albanes D and Virtamo J (1999) Diet and risk of colorectal cancer in a cohort of Finnish men. *Cancer Causes Control* **10**: 387–396

Potter JD (1996) Nutrition and colorectal cancer. *Cancer Causes Control* **7**: 127–146

Rao AV, Janezic SA, Friday D and Kendall CW (1992) Dietary cholesterol enhances the induction and development of colonic preneoplastic lesions in C57BL/6J and BALB/cJ mice treated with azoxymethane. *Cancer Lett* **63**: 249–257

Rastas M, Seppänen R, Knuts L-R, Karvetti R-L and Varo P (eds) (1989). Nutrient composition of foods. (In Finnish with English affiliations.) Publications of the Social Insurence Institution: Helsinki, Finland

Rose DP, Boyar AP and Wynder EL (1986) International comparisons of mortality rates for cancer of the breast, ovary, prostate, and colon, and per capita food consumption. *Cancer* **58**: 2363–2371

Singh PN and Fraser GE (1998) Dietary risk factors for colon cancer in a low-risk population. *Am J Epidemiol* **148**: 761–774

Slattery ML, Caan BJ, Potter JD, Berry TD, Coates A, Duncan D and Edwards SL (1997) Dietary energy sources and colon cancer risk. *Am J Epidemiol* **145**: 199–210

Steinmetz KA and Potter JD (1994) Egg consumption and cancer of the colon and rectum. *Eur J Cancer Prev* **3**: 237–245

Teppo L, Pukkala E and Lehtonen M (1994) Data quality and quality control of a population-based cancer registry. Experience in Finland. *Acta Oncol* **33**: 365–369

Willett WC, Stampfer MJ, Colditz GA, Rosner BA and Speizer FE (1990) Relation of meat, fat, and fiber intake to the risk of colon cancer in a prospective study among women. *N Eng J Med* **323**: 1664–1672

World Cancer Research Fund/American Institute for Cancer Research (1997) Food, nutrition and prevention of cancer: a global perspective. American Institute for Cancer Research: Washington, DC.

World Health Organization (1955) International classification of diseases. Manual of international statistical classification of diseases, injuries, and causes of death. Seventh Revision. World Health Organization: Geneva

© 2001 Cancer Research Campaign
